# Intrarectal Capsazepine Administration Modulates Colonic Mucosal Health in Mice

**DOI:** 10.3390/ijms23179577

**Published:** 2022-08-24

**Authors:** Vibhu Kumar, Vijay Kumar, Kirti Devi, Ajay Kumar, Rehan Khan, Ravindra Pal Singh, Sivasubramanian Rajarammohan, Kanthi Kiran Kondepudi, Kanwaljit Chopra, Mahendra Bishnoi

**Affiliations:** 1 TR(i)P for Health Laboratory, Centre for Excellence in Functional Foods, Department of Food and Nutritional Biotechnology, National Agri-Food Biotechnology Institute (NABI), Sector 81, Mohali 140306, India; 2 University Institute of Pharmaceutical Sciences, Panjab University, Chandigarh 160014, India; 3 Department of Biotechnology, Panjab University, Sector 25, Chandigarh 160014, India; 4 Institute of Nanoscience and Technology (INST), Sector 81, Mohali 140306, India; 5 Department of Microbiology and Cell Science, Cancer and Genetics Research Complex, 2033, Mowry Road, University of Florida, Gainesville, FL 32610, USA; 6 Humboldt Fellow, Klinik für Anästhesiologie, Friedrich-Alexander Universität Erlangen-Nürnberg, Krankanstrasse, 91054 Erlangen, Germany

**Keywords:** capsazepine, mucin, nociceptors, TRPA1, TRPV1

## Abstract

Antagonism of transient receptor potential vanniloid-1 (TRPV1) and desensitization of transient receptor potential ankyrin-1 (TRPA1) nociceptors alleviate inflammatory bowel diseases (IBD)-associated chronic pain. However, there is limited literature available about their role in regulating the mucosal layer, its interaction with host physiology, and luminal microbial community. The present study focuses on the effects’ intra rectal administration of capsazepine (modulator of TRPA1/TRPV1 expressing peptidergic sensory neurons) on colonic mucus production and gut health. We performed histological analysis, gut permeability alteration, gene expression changes, metabolite profiling, and gut microbial abundance in the ileum, colon, and cecum content of these animals. Intra rectal administration of capsazepine modulates TRPA1/TRPV1-positive nociceptors (behavioral pain assays) and resulted in damaged mucosal lining, increased gut permeability, and altered transcriptional profile of genes for goblet cell markers, mucus regulation, immune response, and tight junction proteins. The damage to mucosal lining prevented its role in enterosyne (short chain fatty acids) actions. These results suggest that caution must be exercised before employing TRPA1/TRPV1 modulation as a therapeutic option to alleviate pain caused due to IBD.

## 1. Introduction

Sensory neurons innervating the gastrointestinal tract play a significant role in IBD [1]. The release of neuropeptides, calcitonin gene-related peptide (CGRP), and substance P (SP), from sensory neurons, induce vasodilation and activation of immune cells [2]. Transient receptor potential (TRP) ion channels, particularly Vanilloid-1 (V1) and Ankyrin-1 (A1), have a significant expression in sensory neurons. Recently, efforts to classify colonic sensory neurons using single-cell RNA sequencing analysis suggest a high percentage of TRPA1^+ve^ sensory peptidergic neurons that innervates the colon [3]. Moreover, it has been reported that TRPA1 selectively co-expresses over a larger subset of sensory neurons that express TRPV1 [4] and are found to be involved in symptoms of IBD [1,5] or colonic distension pain in rodents [6]. In the rodent model of dextran sodium sulfate (DSS)-induced colitis, neuronal TRPA1 sensitization in the colon resulted in SP release that contributes to inflammation [1]. In the same study, TRPA1 agonist 2,4,6-trinitrobenzene-sulfonic-acid (TNBS) was used for induction of colitis in mice [1]. Evidence exists on the use of TRPA1 agonists, Allyl isothiocyanate (AITC), and formalin, to induce colitis in rodents [7,8]. Similarly, TRPV1 antagonism has also been proven to attenuate disease severity in mouse and rat models for colitis [9,10]. Given the importance of TRPA1 and TRPV1 nociceptive sensory neurons in colitis-mediated nociception [1,10,11,12,13], the targeted inhibition of these nociceptors can be a promising therapeutic strategy for alleviation of IBD-associated pain.

Capsazepine (CPZ) is a selective inhibitor for TRPV1 channel and have been employed as a treatment to reduce severity of colitis in pre-clinical models [9,10,13]. An intriguing study by Kistner et al. in 2016 proposed an additional mechanism of CPZ. They claimed that CPZ activates TRPA1 in DRG neurons and the rectal administration of CPZ systemically desensitizes TRPA1/TRPV1 expressing peptidergic sensory neurons and attenuates pain and DSS-induced colitis in mice [12]. Though CPZ has been employed as a strategy to ameliorate colitis related pain, its physiological effects on gut health and involvement of its key targets (TRPA1/TRPV1 sensory neurons) still remains elusive.

The intestinal mucus layer protects against mechanical, chemical, and biological attacks and contributes to the maintenance of intestinal homeostasis [14] as an interplay between host and microbe. Alterations like a decrease in the mucin layer can induce gut dysfunction [15]. There is limited evidence available for the effects of CPZ as well as the involvement of TRPA1 and TRPV1 expressing sensory neurons in intestinal mucosal health. Only a short study by Kawabata and group reported that subcutaneous treatment of CPZ antagonized the TRPV1 channel expressed on sensory neurons, which in-turn inhibited the PAR2 mediated gastric mucus secretion [16]. Regarding the involvement of TRPA1 and TRPV1 channel, a single study demonstrated that TRPA1 agonist cinnamaldehyde induces HCO^3−^ secretion in colon, which is essential for mucus release [17]. Recently published work from our laboratory reported that systemic TRPV1 ablation leads to impaired mucus secretion and causes dysbiosis in the gut [18]. Considering the mucus lowering effects of CPZ and importance of TRPA1 and TRPV1 in the gut mucin-immune axis, we hypothesize that using CPZ (for inhibiting or desensitizing TRPV1 and TRPA1 nociceptors) as a strategy for the treatment of IBD-associated pain might impair colonic mucin homeostasis, leading to increased gut permeability and dysbiosis.

Short-chain fatty acids (SCFAs), the enterosyne released from anaerobic bacterial fermentation of dietary substrates (mainly prebiotic containing indigestible carbohydrates) [19,20,21], possess gut modulatory properties. They are significantly involved in the maintenance of colonic mucus production, intestinal barrier integrity, and also have anti-inflammatory and immunomodulatory properties [22,23,24,25]. The association between SCFAs and IBD is well known. Low levels of SCFAs in feces are correlated with an increased risk of IBD [26]. Several studies have reported the use of SCFAs rectal enema in the improvement of IBD symptoms in humans [27,28,29] and rats. In addition, intracolonic infusion of SCFAs is also reported to increase colonic crypt length, mucosal generation, and DNA content in colonocytes [30]. Overall, SCFAs improve colonic mucosal health and play a key role in the protection of the gut against IBDs.

Sensing the significance of TRPA1 and TRPV1 co-expression [4,31], their high abundance in colonic peptidergic sensory neurons, and modulatory effect of CPZ on the activity of these nociceptors, the present study was designed to investigate the impact of intrarectal CPZ administration (through modulation of TRPA1/TRPV1 sensory neurons) on gut mucosal health [12]. In addition, considering the protective effects of SCFAs in the colon, we further sought to explore whether these nociceptive neurons have a modulatory role in the action of SCFAs. These findings will serve to inform considerations on unwanted effects of CPZ (viaTRPA1/TRPV1 modulation) as a treatment strategy for IBD-induced pain.

## 2. Results

### 2.1. Rectal Administration of CPZ Inhibits TRPA1 and TRPV1 Mediated Nociceptive Responses and Modulates the Expression of Peptidergic Genes in DRGs

Rectal administration of CPZ significantly altered TRPA1 associated nociception as evident from reduced AITC-induced eye wipes and elevated tail withdrawal latency time at 4 °C (Figure S1A) as compared to control animals. These effects were constant up to the end of the study (day-14) (Figure S1B). On day-14, the TRPV1-associated nociceptive behavioral assay showed that there was also a significant decrease in capsaicin (selective TRPV1 agonist) induced eye wipes and significant increase in tail withdrawal latency at 42 °C as compared to control group (Figure S1C), suggesting sustained modulation of TRPA1 and TRPV1 expressing sensory neurons. The representative figure from the study of Hockley et al. [3] shows that 49% of TRPA1 expressing colonic sensory neurons belong to mPeptidergic-type-b (mPEPb) class (Figure S1D). CPZ treatment significantly reduced the expression levels of *Spp1*, *Trpa1*, *Th*, and *Calca* genes (Figure S1E) as compared to control animals, which are specifically expressed in the mPEPb class of colonic sensory neurons.

### 2.2. Intrarectal CPZ Administration Negatively Regulates Mucus Homeostasis

**Figure 1:**
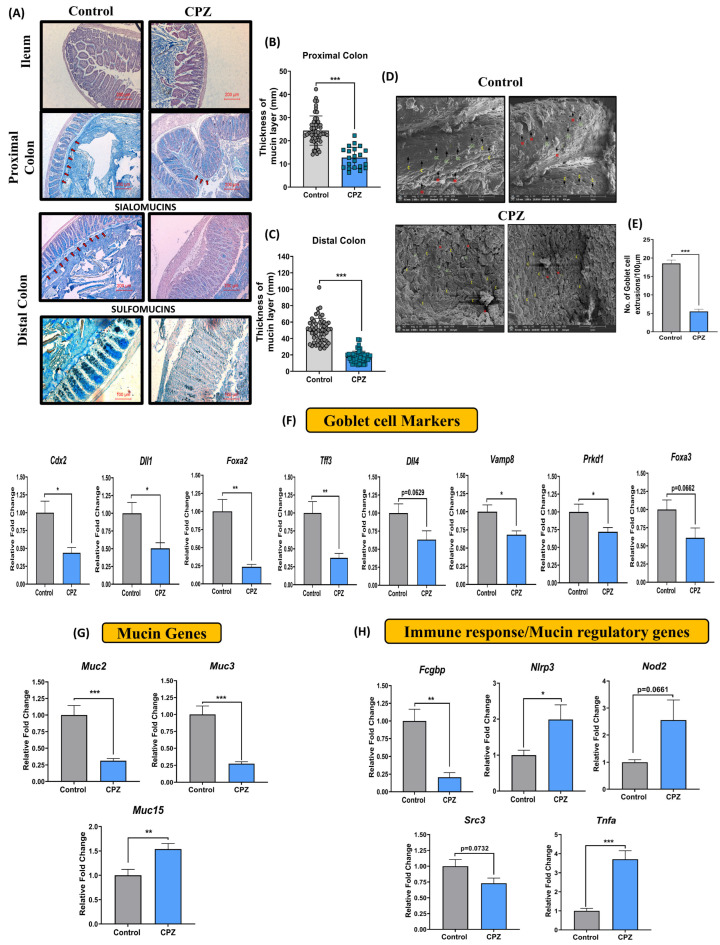
Effect of CPZ rectal administration on colonic mucus production. Histology representative images of (**A**) Hematoxylin and Eosin—Alcian blue staining in ileum, proximal, and distal colon (*n* = 3), High iron diamine—Alcian blue staining in distal colon (*n* = 3), (**B**) thickness of mucus layer in proximal colon, (**C**) thickness of mucus layer in distal colon. (**D**) representative images for SEM of colon tissues and (**E**) number of colonic mucus extrusion (*n* = 3). Gene expression analysis for (**F**) Goblet cell markers, (**G**) mucin genes and (**H**) immune response/mucus regulation in colon (*n* = 6). Mice were divided into two groups—Control (administered vehicle rectally) and CPZ (administered 531 µM CPZ rectally). Treatment was given for two weeks. After sacrifice, colon tissues were fixed in Carnoy’s solution, subjected to serial dehydration with gradient ethanol (25%, 50%, 75%, 90% and twice in 100%) 2 h each followed by clearing in xylene (1 h twice). The colon tissues were embedded in paraffin and 5 µm sections were cut. For staining, the sections were deparaffinized in xylene (5 min, twice) followed by rehydration in gradient ethanol (100% twice, 90%, 70%, 50%, 25% and PBS, 2 min each). The samples were treated with respective stains (H&E: 2 min each, AB: 15 min, HID: 18 h, AB: 30 min) followed by washing (5 min, thrice after each stain) in water. Sections were dehydrated, treated with xylene, and mounted with DPX mounting medium. Microscopy was performed for imaging and mucus thickness was measured using Imagescope. For H&E-AB staining, images were captured at 10× and, for HID-AB staining, images were captured at 20×. The mucus layer in H&E-AB stain tissues is presented with red arrows in the figure. For SEM, colon sections were cut open (lumen was exposed) and fixed in 2.5% glutaraldehyde. Then, the tissues were washed with PBS (15 min, thrice at 4 °C). The tissues were post-fixed in 1% osmium tetroxide for 2 h and again washed with PBS (15 min, thrice at 4 °C). The tissues were then serial dehydrated in acetone (30%, 50%, 70%, 90%, 95%, 100%, 30 min at 4 °C). The samples were air dried and spur-coated with gold (10 nm) images were taken on a scanning electron microscope at 1000×. In SEM image analysis, (M in red) depicts colonic mucus extrusions, (GC in green) depicts goblet cells, and (C in yellow) depicts crypts of the colon. The mucus extrusions were counted using Imagescope. For gene expression studies, RNA was extracted from colon tissues and gene expression was performed for goblet cell markers, mucin genes, and genes involved in immune response and mucus regulation using an Nanostring nCounter multiplex gene expression assay. All data are represented as mean ± SEM. Intergroup variations were assessed using a Student’s unpaired *t*-test. * *p* < 0.05, ** *p* < 0.01, *** *p* < 0.001 versus Control.

Histological analysis of the colon on day 14 revealed that CPZ administration markedly reduced the thickness of the mucus layer in lumen of the proximal and distal colon (Figure 1A–C) as compared to control animals. In addition to this, in distal colon, a significant decrease in goblet cell numbers, as well as alcian blue (AB) and high iron diamine (HID) stain intensity (as a measure of diminished sialomucins and sulfomucins), was observed after CPZ treatment (Figure 1A). Ileum did not show any morphological changes. SEM analysis showed a major decrease in the number of mucus extrusions (M) from goblet cells (GC) and crypts (C) of the colon indicating reduced mucus release after CPZ treatment. In addition, the mucosal architecture with a smooth velvet appearance in the control group was lost upon CPZ treatment (Figure 1D,E). Substantiating these findings, expression levels of genes involved in goblet cell functioning—*Cdx2, Dll1, Foxa2, Tff3, Vamp8,* and *Prkd1*—were downregulated in the distal colon following CPZ administration (Figure 1F). The difference was observable in genes *Dll4* and *Foxa3* but was statistically insignificant (Figure 1F). Genes involved in colonic mucus production—*Muc2* and *Muc3*—were repressed in the distal colon of CPZ-treated animals (Figure 1G). Alternatively, the expression of *Muc15* was significantly elevated (Figure 1G). Another set of genes involved in the immune response—*Nlrp3*, *Nod2*, and *Tnfα*—were significantly upregulated, while *Fcgbp* expression level was reduced with CPZ treatment (Figure 1H).

### 2.3. Rectal Administration of CPZ Increases Intestinal Permeability and Compromises Gut Barrier Function

**Figure 2:**
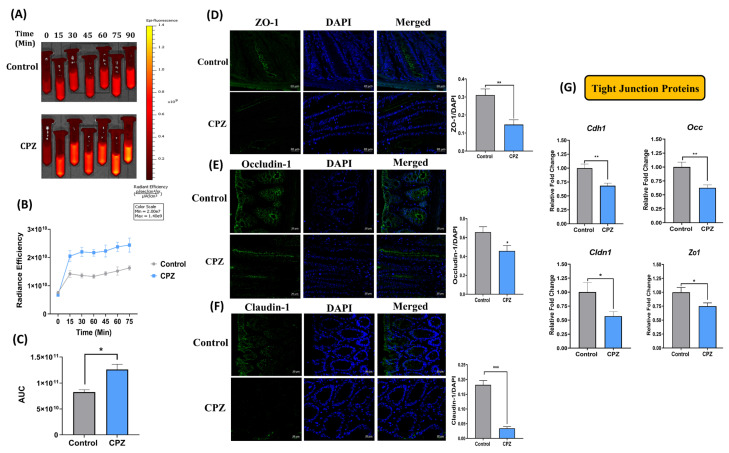
Effect of CPZ rectal administration on intestinal permeability and tight junction proteins. (**A**) in-vitro intestinal permeability—representative images for release of FITC-loaded nanoparticles from colon at different time points (*n* = 3), (**B**) time-based plot for radiance efficiency and (**C**) AUC of the radiance efficiency. Representative images for immunofluorescence in colon tissues for expression of—(**D**) Zona occludens-1 (ZO-1), (**E**) Occludin (OCC), and (**F**) Claudin-1 (CLDN-1) (*n* = 3). Fluorescence intensity was measured using ImageJ, and data are presented after normalization with DAPI; (**G**) gene expression analysis for tight junction proteins. Mice were divided into two groups—Control (administered vehicle rectally) and CPZ (administered 531 µM CPZ rectally). Treatment was given for two weeks. After sacrifice, colon tissues (*n* = 3) were fixed in carnoy fixative, paraffin embedded moulds were prepared, and 5 µm sections were obtained. The sections were subjected to IHC with antigen retrieval using citrate buffer pH 6.0 (90 °C, 60 min) followed by blocking in 5% goat serum in PBS-Tween20, overnight incubation in primary antibodies, secondary antibody treatment for 2 h and DAPI for 30–60 s. High resolution images at 40× magnification were obtained using a confocal microscope, and florescence intensity was measured using ImageJ. For gene expression studies, RNA was extracted from colon tissues, and gene expression was performed for tight junction proteins using a Nanostring nCounter multiplex gene expression assay. All data are represented as mean ± SEM. Intergroup variations were assessed using a Student’s unpaired *t*-test. * *p* < 0.05, ** *p* < 0.01, *** *p* < 0.001 versus Control.

In-vitro intestinal permeability was evaluated by time-dependent fluorescent imaging. In this study, the diffused FITC-loaded nanoparticle samples withdrawn at different time points were imaged and analyzed for membrane barrier function. We have observed intense fluorescent intensity in the CPZ group as compared with the control group (Figure 2A,B). Furthermore, the area under curve (AUC) in CPZ treated animals indicated a higher diffusion rate of FITC-loaded nanoparticles from the colon and suggested a compromised intestinal permeability (Figure 2C). It has also been observed that the expressions of Tight Junction Proteins (TJPs; Zona occludens-1 (ZO-1), Occludin (OCC), and Claudin-1 (CLDN-1)) in colon sections were downregulated in the CPZ group as compared to the control group as indicated by immunohistochemical evaluation (Figure 2D–F). Gene expression studies also indicated a similar trend of reduction in expression of *Cdh, Cldn-1*, *Zo-1*, and *Occ* in the colon of CPZ-treated animals as compared to the control group (Figure 2G).

### 2.4. Rectal CPZ Treatment Alters Mucus Associated Cecal Metabolites and Mucin Glycosylation Enzymes

The GC-MS-based metabolome of cecal content displayed significantly lower levels of mucus-related glycans (fructose, ribose, xylose, xylulose, glucose, and arabinose) and proline, one of the core amino acids of PTS domain in mucin for its structural integrity (Figure S2A). In addition, most of the genes for enzymes involved in mucus glycosylation—*B4galnt-2, C3gnt-1, St3gal4*—were also significantly downregulated in the CPZ group as compared to control. On the other hand, there were also glycosylation genes—*Galnt-1 and Galnt-3*—that were upregulated compared to Control (Figure S2B).

### 2.5. Intrarectal Administration of CPZ Induced Gut Microbiota Changes

**Figure 3:**
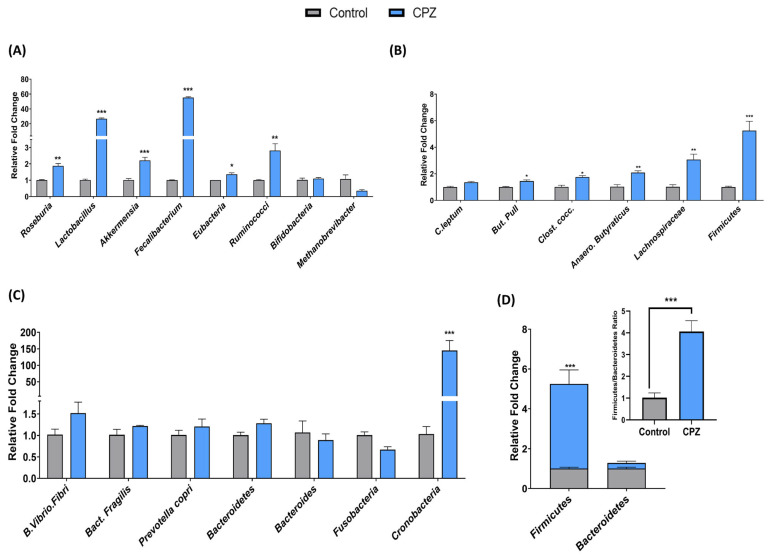
Effect of CPZ rectal administration on gut microbiota. (**A**,**B**) Relative bacterial abundances of Gram^+ve^ bacteria in cecum, (**C**) relative bacterial abundances of Gram^−ve^ bacteria in cecum and (**D**) relative abundance of *firmicutes* and *bacteroidetes* in cecum (*n* = 3). Mice were divided into two groups—Control (administered vehicle rectally) and CPZ (administered 531 µM CPZ rectally). Treatment was given for two weeks. After sacrifice, the cecum content was collected, weighed and bacterial DNA was isolated using a commercial kit. The DNA quantity was measured, and quality was assessed with agarose gel electrophoresis. The DNA was subjected to qRT-PCR based changes in gene expression of various gut bacteria. The data were normalized with total bacteria as internal control and presented in the form of relative fold change. All data are represented as mean ± SEM. Intergroup variations were assessed using a Student’s unpaired *t*-test. * *p* < 0.05, ** *p* < 0.01, *** *p* < 0.001 versus Control.

CPZ treatment increased the abundance of Gram^+ve^ bacteria—*Roseburia, Lactobacillus, Akkermansia, Fecalibacterium, Eubacteria, Ruminococci, Butyricicoccus pullicaecorum, Clostridium coccoides, Anaerostipes butyraticus, Lachnospiraceae,* and *Firmicutes* significantly (Figure 3A,B). However, the abundance of Gram^−ve^ bacteria–*Butyrivibrio fibrisolvens, Bacteroides fragilis, Prevotella copri, Bacteroidetes, Bacteroides,* and *Fusobacteria* remained unaltered with CPZ treatment (Figure 3C). Only *Cronobacteria* was increased in a significant manner as compared to the control group (Figure 3C). The *Firmicutes–Bacteroides* ratio was significantly higher in CPZ-treated animals (Figure 3D). Furthermore, to evaluate host-independent effects of CPZ on gut microbiota, fresh feces from normal mice were subjected to in-vitro fermentation in the presence of CPZ for 48 h. qRT-PCR analysis revealed that abundances of Gram^−ve^ bacteria—*Butyrivibrio fibrisolvens, Prevotella, Bacteroidetes, Fusobacteria, Bacteroides, Cronobacteria,* and *Firmicutes* were significantly reduced in CPZ treated animals as compared to Control (Figure S3A). Out of Gram^+ve^ bacteria, the abundance of *Roseburia* was reduced, and the abundance of *Lactobacillus* was elevated with CPZ treatment as compared to control (Figure S3B). No effect was observed on other Gram^+ve^ genera–*Akkermansia, Fecalibacterium, Eubacteria, Ruminococci, and Anaerostipes butyraticus* in the presence of CPZ. 

### 2.6. Rectal Co-Administration of SCFA Mix Fails to Prevent CPZ—Induced Compromise in Colonic Mucin Homeostasis

**Figure 4:**
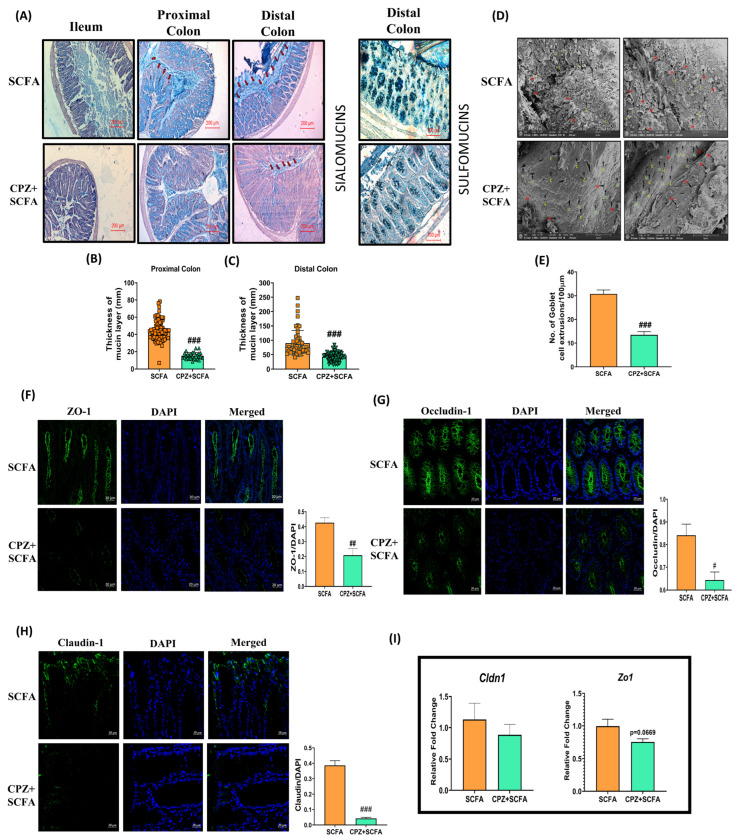
Effect of rectal co-administration of SCFAs on CPZ-induced alterations in colonic mucus production and tight junction proteins. Histology representative images of (**A**) Hematoxylin and Eosin—Alcian blue staining in ileum, proximal, and distal colon (*n* = 3), High iron diamine—Alcian blue staining in distal colon (*n* = 3), (**B**) thickness of mucus layer in proximal colon, (**C**) thickness of mucus layer in distal colon, (**D**) representative images for SEM of colon tissues, and (**E**) number of colonic mucus extrusion (*n* = 3). Representative images for immunofluorescence in colon tissues for expression of—(**F**) Zona occludens-1 (ZO-1), (**G**) Occludin (OCC), and (**H**) Claudin-1 (CLDN-1) along with their fluorescence intensities (*n* = 3). Fluorescence intensity was measured using ImageJ and data are presented after normalization with DAPI, (**I**) gene expression analysis for tight junction proteins (*n* = 6). For Histology, mice were divided into two groups (*n* = 6): SCFA (treated with rectal administration 200µL SCFA mix [180 mM Acetic acid + 60 mM Propionic acid + 60 mM Butyric acid for 14 days]) and CPZ + SCFA (initially treated with 531 µM CPZ twice daily for seven days followed by once daily dose of 531 µM CPZ along with rectal administration of SCFA mix for the next seven days). For staining, the sections were deparaffinized in xylene (5 min, twice) followed by rehydration in gradient ethanol (100% twice, 90%, 70%, 50%, 25% and PBS, 2 min each). The samples were treated with respective stains (H&E: 2 min each, AB: 15 min, HID: 24 h) followed by washing (5 min, thrice after each stain) in water. Sections were dehydrated, treated with xylene and mounted with DPX mounting medium. Microscopy was performed for imaging and mucus thickness was measured using Imagescope. For H&E-AB staining, images were captured at 10× and for HID-AB staining, images were captured at 20×. The mucus layer in H&E-AB stain tissues is presented with red arrows in the figure. For SEM, colon sections were cut open (lumen was exposed) and fixed in 2.5% glutaraldehyde. Then, the tissues were washed with PBS (15 min, thrice at 4 °C). The tissues were post-fixed in 1%osmium tetroxide for 2 h and again washed with PBS (15 min, thrice at 4 °C). The tissues were then serial dehydrated in acetone (30%, 50%, 70%, 90%, 95%, 100%, 30 min at 4 °C). The samples were air dried and spur-coated with gold (10 nm) and images were taken on a scanning electron microscope at 1000×. In SEM image analysis, (M in red) depicts colonic mucus extrusions, (GC in green) depicts goblet cells, and (C in yellow) depicts crypts of the colon. The mucus extrusions were counted using Imagescope (version 12.1.0.5029, Leica Biosystems, Wetzlar, Germany). For IHC, colon tissues (*n* = 3) were fixed in carnoy fixative, paraffin embedded moulds were prepared, and 5 µm sections were obtained. The sections were subjected to IHC with antigen retrieval using citrate buffer pH 6.0 (90 °C, 60 min) followed by blocking in 5% goat serum in PBS-Tween20, overnight incubation in primary antibodies, secondary antibody treatment for 2 h and DAPI for 30–60 s. High resolution images were obtained at 40× magnification using confocal microscope and florescence intensity were measured using ImageJ (version-1.52, NIH, Bethesda, MD, USA). For gene expression studies, RNA was extracted from colon tissues, and gene expression was performed for tight junction proteins using a Nanostring nCounter multiplex gene expression assay. All data are represented as mean ± SEM. Intergroup variations were assessed using the Student’s unpaired *t*-test. ^#^ *p* < 0.05, ^##^ *p* < 0.01, ^###^ *p* < 0.001 versus SCFA.

Rectal administration of SCFA mix *per se* increased the thickness of the mucus layer, as well as sialomucins and sulfomucins content in goblet cells. However, its co-administration with CPZ is unable to prevent the CPZ-mediated reduction in goblet cells’ differentiation and mucus production (Figure 4A–C). Colon tissues from SCFA *per se* treated mice displayed a significant increase in mucus extrusions (M) from goblet cells (GC) and crypts (C) of colon, but a minimal effect was observed upon their co-administration with CPZ. In addition, SCFA co-administration was unable to reverse the CPZ-induced morphological alteration in mucosal architecture (Figure 4D,E). Furthermore, the SCFA mix intervention increased the expression of TJPs—ZO-1, OCC, and CLDN-1—but CPZ-induced downregulation in these proteins was unaffected with SCFA mix co-administration (Figure 4F–H). Similarly, qRT-PCR results of *Cldn-1* and *Zo-1* displayed a similar trend but were statistically non-significant (Figure 4I).

### 2.7. Principal Component and Correlation Analysis

The PC analysis revealed that the control group clustered separately from the CPZ group indicating a distinctly altered gut microbial abundance, metabolites, and host gene expression profile of the CPZ group from that of the control group (Figure S4A–C). Additionally, most of the parameters assessed displayed a negative correlation between themselves and other parameters between the Control and CPZ groups (Figure S4D). Similarly, the PCA of SCFA and CPZ-SCFA groups showed distinct clustering of the groups based on gut microbiota and metabolites (Figure S5A,B). However, in the PCA based on host gene expressions, the groups showed only a slight dissimilarity (Figure S5C), indicating that CPZ treatment inhibits the beneficial effects of SCFAs in the colon. In addition, the pattern in the correlation matrix displayed a negative correlation in most of the parameters between both of the groups (Figure S5D).

## 3. Discussion

Nociceptors, in particular TRPA1 and TRPV1 expressing peptidergic sensory neurons, regulate epithelial cell function (including goblet cells) owing to their involvement in neurogenic inflammation [12,32]. There are numerous reports delineating their role in various gut-related diseases [1,9,13,33,34]. The release of these mediators and changes in mucus production in the gut epithelial layer, in response to nociceptor activation, have an ability to affect the microbial population cyclically. Moreover, there is literature describing the reciprocal relationship between the gut microbial population and its role in mucin secretion, which influences not only mucus layer formation but also its regulation [35,36]. In our study, rectal administration of CPZ caused a loss of mucin layer, prominently in the colon. Both forms of mucin (sialomucin and sulfomucin) were found to be significantly decreased. According to the findings of a brief investigation by Kawabata et al., our observation of decreased mucus secretion is strongly corroborated. They demonstrated that subcutaneous administration of CPZ antagonizes the TRPV1 channel expressed on sensory neurons and reduces the PAR-2 mediated gastric mucus secretion [16]. In our previous studies, we also found that capsaicin induced activation of TRPV1 promotes mucus secretion in the gut and resiniferatoxin-mediated chemodenervation of the TRPV1 expressing sensory neurons severely impairs gut mucus production and promotes gut dysbiosis [18,37] Moreover, Kistner et al. demonstrated that CPZ rectal administration desensitizes TRPA1/TRPV1 expressing sensory neurons [12]. With support of available literature, we argue that CPZ-induced alterations in colonic mucosal health will be mediated through TRPV1 and TRPV1 channels. Even if CPZ desensitizes only TRPA1, there is a possibility that both TRPA1 and TRPV1 are not functional now as they both co-express on the same neuron [4]. Hence, our argument that CPZ induced changes are mediated through A1, and V1 is a possibility. We did not do knockout studies, but we performed nociceptive behavioral assays using agonists specific to these channels (AITC and Capsaicin) and temperature mediated pain behavior selective to these channels. Our observations of loss of TRPA1 and TRPV1 mediated nociceptive responses (both chemical and temperature) are consistent with the study by Kistner et al. [12]. Hence, there is a strong possibility that the CPZ actions are mediated through TRPA1 and TRPV1 channels. The expression of these channels can still be there due to their presence in any other cell type. Though the study presents the idea of CPZ-induced modulation of TRPA1 and TRPV1, the specific interactions and mechanisms are still in infancy and an interesting area for further research. Mice treated with CPZ had significantly enhanced gut permeability as studied by the in-vitro nanoparticle diffusion approach through the colon. In CPZ-treated animals, both protein and gene expression for tight-junction proteins (TJP)s were significantly decreased suggesting both translational and transcriptional decrease in tight junction morphology. These findings are of significance because CPZ treatment prevented the SCFA effect in these animals [25,38,39,40], hence suggesting that the intact mucin layer is important for the effects of healthy microbial metabolites in the host gut.

*Muc3* and *Muc2* genes were significantly decreased in CPZ treated animals. In studies on *Muc2* knockout animals, increased intestinal permeability has been shown to trigger a cascade of events leading to secondary organ inflammation and translocation of bacterial metabolites and bacteria *per se* leading to sepsis [41,42]. Different goblet cell markers (*Cdx2, Dll1, Foxa2, Tff3, Dll4, Vamp8, Prkd1, and Foxa3*) and *Fcgbp* were significantly decreased in the CPZ group. A decrease in *Muc3* (a major transmembrane mucin present on the N-terminal of extracellular mucin domain tip of the enterocyte microvilli) points to compromised cell protection (due to its role in densely glycosylated glycocalyx) and altered signal transduction pathways associated with the microbe: host interactions [43]. In contrast, another cell surface associated with *Muc15* was significantly higher in the colon of CPZ treated mice and has a role in carcinogenesis and protein metabolism [44]. Alteration in levels of these mucin secreting and transmembrane genes is suggestive of compromised mucin homeostasis, overall making the animals in the CPZ group prone to disorders. This is reflected in the increase in the expression of genes related to immune response activation (*Nlrp3, Nod2, Tnfα*) [45,46]. *Nlrp3* and *Tnfα* are very well known for their role in the disruption of intestinal permeability [25,47,48,49]. Thereby, the increased expression of these inflammatory genes can be directly related to increased intestinal permeability in CPZ treated animals. In contrast, the *Nod2* gene has been reported to be associated with improvement in colonic mucus production, intestinal permeability, and inflammation [50,51]. The increased expression levels of *Nod2* in our findings might suggest a compensatory reflex mechanism to reverse the colonic alterations developed due to CPZ treatment.

The mucin layer is a source of nutrition and provider of attachment sites for gut microbes [52]. In the absence of the mucin layer (decrease in glycosylation marker genes) in CPZ treated mice, colonic bacteria are deprived of both nutrients and their attachment sites forcing them to rely more on diet-extracted nutrition. A major part of the diet comprises plant dietary fiber, i.e., high molecular weight carbohydrates (polysaccharides). Metabolite analysis in cecal samples showed a decrease in the monomeric metabolites of these polysaccharides in CPZ treated animals indicating greater utilization of these by local microbiota as a nutrient source. During bacterial profiling in these samples, the increase in the (a) levels of Firmicutes and (b) ratio of Firmicutes/Bacteroidetes suggests increased extraction of energy from the dietary sources [53]. Intestinal mucus requires the presence of bacteria for its function. Hence, the significant increase in good commensal bacteria (*Lactobacillus, Fecalibacterium,* and *Akkermensia,* etc.) as well as SCFA producing bacteria—*Roseburia, Lactobacillus, Fecalibacterium, Eubacteria, Ruminococci, Butyricicoccus pullicaecorum, Clostridium coccoides, Anaerostipes butyraticus,* etc. [54] in CPZ treated animals suggests their adaptive response towards reduction in the mucin layer to support and maintain colonic mucosal health as we did not see any significant effect of CPZ in most of the individual groups of these bacteria during in-vitro fecal batch fermentation. Overall, the PC and correlation analysis indicate that (a) Rectal administration of CPZ caused considerable changes in overall microbiota, metabolite, and gene expression profile and (b) prevented beneficial effects of SCFAs. However, it is still unclear whether the altering effects on colonic mucosa were attributed selectively to TRPA1 and TRPV1 nociceptive sensory neurons modulation or the CPZ treatment or both. Moreover, the reduced abundance of some gut bacteria during in-vitro fermentation experiments points toward the direct effect of CPZ on the gut microbial community. Thus, using an additional selective approach for the non-functionalization of TRPA1 and TRPV1 might give a clearer picture of the exact role of these nociceptors on colonic mucosal alterations. We have planned for in-vivo follow-up studies in the mouse model of IBD (colitis) (using global and tissue-specific TRPA1 and TRPV1 knock out animals or TRPA1/V1 double knock out and, TRPA1/TRPV1 silencing approach in primary cells) to establish an exact role of neuronal nociceptive TRPA1 and TRPV1 channels in colonic mucosal alterations, especially in IBD conditions. Additionally, the number of animals used per group are limited (*n* = 5–6). In our future studies with IBD model (colitis), we will address this.

## 4. Materials and Methods

### 4.1. Animals

Six–eight weeks-old male C57BL/6J mice (25–30 g) (*n* = 24), procured from IMTech Center for Animal Resources and Experimentation (iCARE; 55/GO/Re/Rc/Bi/Bt/S/99/CPCSEA), Chandigarh, India, and housed (temperature, 24 ± 2 °C; humidity, 55–65%, 12 h light-dark cycle) in Animal Experimentation Facility (2039/GO/RBi/S/18/CPCSEA), National Agri-Food Biotechnology Institute (NABI), Mohali, India. Animals were provided with water and a normal pellet diet (Hylasco Biotechnology Pvt. Ltd., Hyderabad, India) *ad libitum.* The study was conducted according to the guidelines of the Declaration of Helsinki, and study protocol was approved by the Institutional Animal Ethics Committee (IAEC) of NABI (Approval No. NABI/2039/CPCSEA/IAEC/2019/16). Committee for the Purpose of Control and Supervision on Experiments on Animals (CPCSEA) guidelines on the use and care of experimental animals were followed throughout the experiment.

### 4.2. Intrarectal Administration of CPZ, TRPA1 and TRPV1-Mediated Nociceptive Behavioral Confirmatory Tests and SCFA Mix Treatment

**Figure 5:**
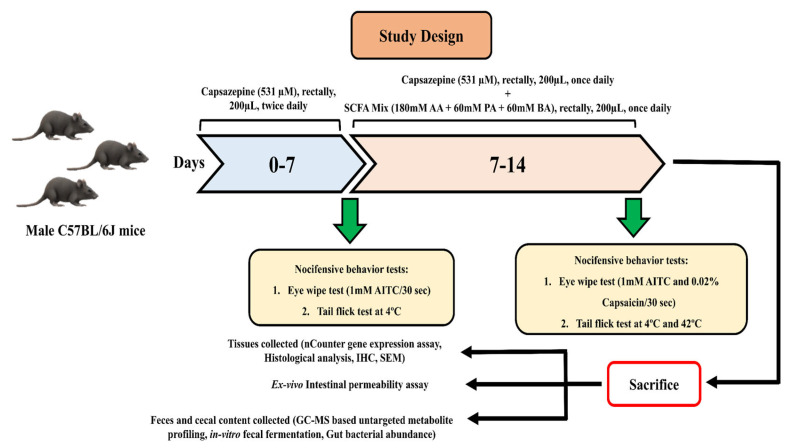
Animal experiment plan.

After 1 week of acclimatization, animals were divided into 4 groups—Control, Capsazepine (CPZ), SCFA (Short-chain fatty acids), and CPZ + SCFA (*n* = 6 each housed in one cage). Intrarectal administration of CPZ was done as per previously described method with slight modification [12]. Rectal administration of 531 µM Capsazepine (CPZ) (200 µL) (Cat. No. C191-25MG, Sigma-Aldrich, St. Louis, MO, USA) enemas was given to 12 animals twice daily for seven days followed by once-daily administration for the next seven days for its maintenance under brief isoflurane anesthesia. Control animals received 200 µL PBS rectally as the vehicle. Modulation of TRPA1 activity was confirmed by a loss of nociceptive physiological responses after 7 days using tail-flick test and eye wipe test as a primary measure. For the tail-flick test, the tip of the tail of mice was dipped in cold water (4 °C) and the time taken to withdraw the tail was noted as a pain response (cut-off time of 30 s). Experiments were repeated 3 times/animal, at intervals of 5 min. For the eye-wipe test, 1 mM AITC solution (Cat. No. 377430; Sigma-Aldrich, St. Louis, MO, USA) was instilled into the eye of the animal, and the number of wipes was recorded for 30 s. After 7 days, the animals receiving CPZ were divided into two groups—one group (CPZ + SCFA) received a 200 µL SCFA mix (180 mM Acetic acid + 60 mM Propionic acid + 60 mM Butyric acid (neutralized to pH 6.5); Cat. No. A6283, 402907, B103500; Sigma-Aldrich, USA, St. Louis, MO, USA) rectally, once a day along with once-daily dose of 200 µL of 531 µM CPZ rectal administration. The dose and composition of SCFA was decided as per previously published literature and a physiological ratio of SCFAs present in the gastrointestinal tract [38,39,55,56,57,58]. The other group (CPZ) only received once-daily administration of 200 µL 531 µM CPZ as a maintenance dose for the next 7 days. Simultaneously, another set of animals was also receiving rectal administration of 200 µL SCFA mix (SCFA group), once a day alone as respective treatment control. The dose and route of administration of SCFA mix was based on previous studies [39,55,59]. At the termination of the study, TRPA1-associated nociceptive behavioral assays (tail-flick test and eye-wipe test) were performed again for the confirmation of the loss of TRPA1 associated nociceptive responses till the end of the study (Day-14). On the same day, TRPV1 associated behavioral tests were also performed. Selective TRPV1 agonist induced eye-wipe test (0.02% *w*/*v* capsaicin solution (Cat. No. 360376; Sigma Aldrich, St. Louis, MO, USA) and tail-flick test (in water kept at 42 °C) were employed for the confirmation of loss of TRPV1 activity. Replicates for each animal were taken using both eyes separately. The plan of experiment is explained in Figure 5.

### 4.3. In-Vitro Gut Permeability Assay Using FITC Loaded Solid-Lipid Nanoparticle

#### 4.3.1. Formulation of FITC Loaded Solid-Lipid Nanoparticle

The fluorescent nanoparticle was prepared according to Ahmad et al. [60]. Briefly, 140 mg of glyceryl monostearate (GMS) (Cat. No. G0085; TCI Chemicals, Tokyo, Japan) and 5 mg of fluorescein isothiocyanate (FITC) (Cat. No. F0784; TCI Chemicals, Tokyo, Japan) were melted above 10 °C of their melting points. The aqueous surfactant PF-127 (Cat. No. P2443; Sigma Aldrich, St. Louis, MO, USA) 2% *w*/*v* (200 mg) was dissolved in 10 mL of distilled water at the same temperature. The aqueous phase was incorporated drop-by-drop into the oil phase over constant stirring of 1500 rpm and kept for 1 h, followed by sonication using a probe sonicator for 10 min (5 s each on and off-cycle at 40 Hz frequency). FITC-nanoparticles were lyophilized and dispersed in milli-Q water before administration.

#### 4.3.2. In-Vitro Gut Permeability Assay

Determination of colon permeability was assessed as a previously reported method [61] with some modification. In brief, the distal colon (2 cm) was excised and carefully flushed with normal saline, after that one end of the colon was ligated with the silk thread and 70 µL of FITC loaded nanoparticles were filled into the colon and the other end of the colon was also secured with silk thread. This assembly was then placed in a beaker filled with 150 mL of PBS buffer (pH 7.4), maintained at 37 °C, and stirred at mild constant RPM (200 rpm). In addition, 1 mL of sample was withdrawn at pre-defined time intervals (0, 15, 30, 45, 60, 75, and 90 min) and replaced with fresh 1 mL of PBS to maintain sink condition. The withdrawn samples were analyzed and imaged for fluorescent intensity by using IVIS Lumina Series-III (Perkin Elmer, Waltham, MA, USA) and analyzed using living image 5.0 software (Perkin Elmer, Waltham, MA, USA).

### 4.4. Gene Expression Analysis in DRGs and NanoString nCounter Multiplex Gene Expression Assay

Total RNA was extracted from dorsal root ganglion (DRGs) using the TRIzol RNA extraction reagent-based method. Isolated RNA was transcribed into cDNA using the Revert Aid cDNA synthesis kit (Cat. No. K1622; Thermo Fischer Scientific, Waltham, MA, USA). The synthesized cDNA was then used to perform gene expression of specific markers of peptidergic sensory neurons [3] by qRT-PCR using SYBR Green (Cat. No. L001752; Bio-Rad Laboratories, Hercules, CA, USA). The conditions were at 95 °C for 2 min, followed by 40 cycles of annealing and elongation at 60 °C for 30 s and denaturation at 95 °C for 5 s. Analysis of relative change in gene expression was done using the 2^−ΔΔCt^ method [62]. Ct values were normalized to the β-actin (housekeeping) gene, and values were expressed in the terms of fold change with reference to control.

For transcriptional studies in colons, a customized Nanostring nCounter GX CodeSet gene expression panel (ILS_Bishnoi/C8788X1; NanoString Technologies, Seattle, WA, USA), comprising genes of goblet cell differentiation (*Cdx2*, *Dll1, Dll4, Foxa1, Foxa2, Foxa3, Klf1, Prkd1, Tff3, Vamp8*)/tight junction protein (TJPs) (*Cdh1, Cldn1, Cldn4, Occ, Zo1, Zo2*)/mucin production (*Muc15, Muc2, Muc20, Muc3, Muc4, Muc5b*)/glycosylation enzyme (*B4galnt1, B4galnt2, C1galt1, C3gnt1, Fut2, Galnt1, Galnt2, Galnt3, St3gal4*)/immune response (*Defb2, Dmbt1, Fcgbp, Nlrp3, Nod2, Reg3g, Src3, Tnfa, Xbp1)/*4 housekeeping genes (*Actb, B2m, Gapdh, Ubc)* was used. Furthermore, 50 ng of extracted RNA sample were hybridized with unique probes using the NanoString nCounter prep station and placed into the cartridge (NanoString Technologies, Seattle, WA, USA). A NanoString nCounter digital analyzer (NanoString Technologies, Seattle, WA, USA) was employed for the detection and counting of hybridized probes. The data were analyzed using nSolver 4.0 software (NanoString Technologies, Seattle, WA, USA).

### 4.5. Histology and Immunohistochemistry Analysis

Ileum, proximal and distal colon were fixed in Carnoy’s solution (60% ethanol + 30% chloroform + 10% glacial acetic acid) and stored in the same until processed. Fixed tissues were serially dehydrated, cleared, and embedded using gradient ethanol, xylene, and molten paraffin, respectively. In addition, 5-µm-thick sections were prepared, deparaffinized in xylene, and rehydrated in gradient ethanol. For staining, Hematoxylin-Eosin (H&E) (Cat. No. S034, S007; Hi-media Laboratories, Mumbai, India) and Alcian blue (AB) (Cat. No. B8438; Hi-media Laboratories, Mumbai, India) were used for 30 s, 15 s, and 15 min, respectively. Slides were mounted using DPX mounting medium (Cat. No. GRM655; Hi-media Laboratories, Mumbai, India). Images were captured at 10× on a bright-field microscope (Leica CTR6, Leica Biosystems, Wetzlar, Germany), and the thickness of the mucus layer was measured using Imagescope (Version 12.1.0.5029, Leica Biosystems, Wetzlar, Germany) manually. For high iron diamine (HID) staining for sulfomucins, rehydrated tissue sections were stained with HID solution for 18 h at room temperature in the dark followed by washing with distilled water 3 times. The sections were then stained with alcian blue for 30 min, washed 3 times with distilled water, and mounted with DPX mounting medium. Images were captured at 20× on the bright-field microscope (Leica CTR6, Leica Biosystems, Wetzlar, Germany).

For immunohistochemistry, 5-µm rehydrated sections were treated for antigen retrieval using citrate buffer (pH 6.0), blocked in 5% goat serum for 1 h followed by incubation in primary antibodies, Zona occludens-1 (*Zo1*)- (1:200 prepared in 2.5% goat serum, Cat. No. ab216880 Abcam, Cambridge, United Kingdom), Occludin (*Occ*) (1:200 prepared in 2.5% goat serum, Cat. No. ab222691 Abcam, Cambridge, United Kingdom), Claudin-1 (*Cldn-1*) (1:200 prepared in 2.5% goat serum, Cat. No. ab15098; Abcam, Cambridge, United Kingdom) for overnight at 4 °C. Finally, the sections were incubated with Alexa fluor 488 (1:1000 prepared in goat serum; Cell Signaling Technology, Danvers, MA, USA) secondary antibody for 2 h at room temperature. DAPI stain was used to stain nuclei. Images were taken under a confocal microscope (Carl Zeiss LSM-880, Carl Zeiss, Oberkochen, Germany) and all the images were captured at 40x magnification. Images were analyzed using ImageJ software (Public domain software, version 1.52, NIH, Bethesda, MD, USA).

### 4.6. Scanning Electron Microscopy

For Scanning Electron Microscopic (SEM) analysis, small fragments of distal colon were fixed in 2.5% glutaraldehyde solution (Cat. No. G5882; Sigma Aldrich, St. Louis, MO, USA) in PBS (pH 7.4) for 6 h at 4 °C. The tissues were washed with PBS, post-fixed in 1% osmium tetroxide (Cat. No. 201030; Sigma Aldrich, St. Louis, MO, USA) in PBS (pH 7.4) for 2 h at 4 °C. Tissues were washed with PBS and dehydrated through the serial application of increasing concentration of acetone (Cat. No. 20003; SDFCL, Chennai, India) viz. 30%, 50%, 70%, 90%, 95%, 100% to remove water at 4 °C for a 30 min period. Samples were air-dried and spur-coated with gold (10 nm). Images were acquired using Apreo S SEM (Thermo Fischer Scientific, Waltham, MA, USA).

### 4.7. Analysis of Microbial Abundance in Cecal Content

DNA was isolated from 100 mg cecal content, using Nucleospin DNA Stool Genomic DNA Purification Kit (Cat. No. 740472; Macherey-Nagel, Düren, Germany), as per the manufacturer’s instruction. DNA was quantified on a spectrophotometer (Nanodrop, Thermo Scientific, Waltham, MA, USA) and run on 0.8% agarose gel electrophoresis for integrity. qRT-PCR was performed as per the protocol detailed in Section 4.4 using bacterial primers (Table 1). Analysis of relative change in the abundance was calculated using the 2^−ΔΔCt^ method [62]. Ct values were normalized to Total Bacteria (Internal control), and values were expressed in the terms of fold change with reference to control.

### 4.8. Untargeted Metabolome Profiling of Cecal Content

Gas chromatography-mass spectrophotometry (GC-MS) was employed for metabolite analysis. Briefly, 80 mg of cecal content was treated with methanol, chloroform, and water (in a 2.5:1:1 ratio) at 4 °C, overnight with shaking. Samples were centrifuged (3000 rpm) and the supernatant was lyophilized. For derivatization, lyophilized samples were treated with 90 µL N, O-bis(trimethylsilyl)tri-fluoroacetamide (BSTFA), 1% trimethylchlorosilane (TMCS) (Cat. No. B-023; Merck &Co., Kenilworth, NJ, USA), and 30 μL of pyridine (Cat. No. 029707; Central Drug House, New Delhi, India) and incubated for 4 h at 70 °C. The samples were diluted up to 500 µL with dichloromethane (DCM) (Cat. No. 508705; Central Drug House, New Delhi, India), and 5 µL were injected into GC-MS through an automatic sampler. An Agilent 7870A chromatograph (Agilent technologies, Santa Clara, CA, USA) equipped with an Agilent triple-quadrupole 7000 mass spectrometer (Agilent technologies, Santa Clara, CA, USA) was employed for performing analysis. A DB-5 column (Agilent, 60 m × 250 μm ID × 0.25 μm) was used for the separation of derivatized molecules before reaching the MS chamber for their efficient identification. The column oven temperature was initially set at 80 °C for 2 min, then increased to 140 °C at 10 °C/min, and then increased from 140 to 250 °C at 5 °C/min. The helium carrier gas flow was set at 1.5 mL/min in spitless mode. For MS, the source temperature was set at 230 °C, electron ionization was set at −70 eV, the scan range was kept at 30–500 *m*/*z,* the gain factor was set at 10, and thermal auxiliary temperatures 1 and 2 were set at 150 and 280 °C, respectively. Mass fragmentation of each peak of the MS spectrum was matched with the NIST-MS library (version 2.0) for identifying metabolites and were reported in the form of percent change.

### 4.9. In Vitro Mouse Fecal Batch Fermentation with CPZ and Relative Bacterial Abundances

Slurry from freshly collected fecal samples (10% *w*/*v*) was prepared in 1X PBS containing cysteine with homogenization. Fecal batch fermentation was done with 531 µM CPZ and glucose (1% *w*/*v*) (Cat. No. MB-037; Himedia Laboratories, Mumbai, India) for 48 h. Basal growth media of the following composition were used (composition (g/L) peptone water 2 g/L, yeast extract 2 g/L, NaCl 0.1 g/L, K_2_HPO_4_ 0.04 g/L, KH_2_PO_4_ 0.04 g/L, MgSO_4_·7H_2_O 0.01 g/L, CaCl_2_·6H_2_O 0.01 g/L, NaHCO_3_ 2 g/L, Tween 80 2 mL, hemin 0.02 g/L, vitamin k1 10 mL, cysteine HCl 0.5 g/L, pH 7.0) (Himedia Laboratories, Mumbai India). Initial pH was maintained at 7. Bacterial DNA extraction was done after 48 h of fermentation using a Nucleospin DNA Stool Genomic DNA Purification Kit (Macherey-Nagel, Duren, Germany), and the relative abundance of selected gut bacteria was determined by qRT-PCR similar to Section 4.7.

### 4.10. Statistics

Data were analyzed using Graphpad Prism 8 software (Graphpad, San Diego, CA, USA). All the data are presented as mean ± SEM. Student’s unpaired *t*-test was employed to assess the statistical differences between the groups. *p* < 0.05 was considered significant in all data. The principal component analysis was carried out using the adegenet (version 1.3-1) package in R [63], while the correlation analysis (using Pearson correlation coefficient) was carried out using the corrplot package in R. The PCA and correlation analysis was carried out using the gut microbial abundance, metabolite levels, and gene expression levels of the different animal groups viz. Control, CPZ, SCFA, and CPZ-SCFA.

## 5. Conclusions

**Figure 6:**
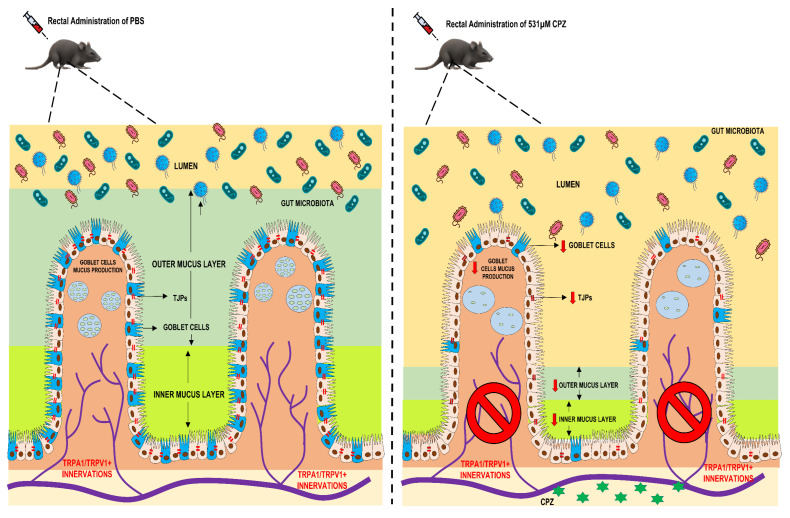
Representative image for overall effects of CPZ-induced alterations in colonic mucus production and gut health.

In conclusion, the present study suggests the significance of CPZ treatment for pain and its unwanted effects (possibly via TRPA1/TRPV1 modulation) on mucosal health (Figure 6). However, it is still not sufficiently clear whether side effects presented here are detrimental (mucous layer loss) or beneficial (adaptive response of increase in the gut microbial ecosystem). These studies are of potential clinical applications as TRPA1/TRPV1 antagonism and desensitization are marked as promising therapeutic approaches for the alleviation of pain associated with IBD and caution may be exercised before employing these approaches as a therapeutic option for the same. Following our evidence presented here, we suggest further exploration into the role of nociceptors in mucin health and host: microbe interactions for better understanding of side effect profiles of these approaches before taking them to clinical practice.

## Figures and Tables

**Table 1:** List of bacterial primers.

Sr. No.	Bacterial Primer	Forward Sequence	Reverse Sequence
1.	*Total Bacteria*	ACTCCTACGGGAGGCAGCAGT	ATTACCGCGGCTGCTGGC
2.	*Butyrivibrio fibrisolvens*	CTAACACATGCAAGTCGAACG	CCGTGTCTCAGTCCCAAT
3.	*Bacteroides fragilis*	GGCGCACGGGTGAGTAACA	CAATATTCCTCACTGCTGC
4.	*Prevotella copri*	CCGGACTCCTGCCCCTGCAA	GTTGCGCCAGGCACTGCGAT
5.	*Clostridium leptum*	CCGCATAAGACCTCAGTACCGC	GGGATTTGCTTGCCTTCACAGGG
6.	*Clostridium coccoides*	ACTCCTACGGGAGGCAGC	GCTTCTTAGTCARGTACCG
7.	*Lactobacillus*	CACCGCTACACATGGAG	AGCAGTAGGGAATCTTCCA
8.	*Bacteroides*	TCCTACGGGAGGCAGCAGT	CAATCGGAGTTCTTCGTG
9.	*Bifidobacterium*	TCGCGTCYGGTGTGAAAG	CCACATCCAGCRTCCAC
10.	*Akkermansia muciniphila*	CAGCACGTGAAGGTGGGGAC	CCTTGCGGTTGGCTTCAGAT
11.	*Butyricicoccus pullicaecorum*	AGTACGGCCGCAAGGTTGAAA	CTGCCATTGTAGTACGTGTG
12.	*Anaerostipes butyraticus*	CACCATGTCATTTACTCAAGAATATCAGA	TTATTTGTTAGATCTTCTCCAGATGTTAGC
13.	*Roseburia* spp.	GCGGTRCGGCAAGTCTGA	CCTCCGACACTCTAGTMCGA
14.	*Fecalibacterium*	GGAGGAAGAAGGTCTTCGG	AATTCCGCCTACCTCTGCACT
15.	*Ruminococci*	GGCGGCYTRCTGGGCTTT	CCAGGTGGATWACTTATTGTGTTAA
16.	*Eubacteria*	ACTCCTACGGGAGGCAGCAG	ATTACCGCGGCTGCTGG
17.	*Methanobrevibacter*	CCGGGTATCTAATCCGGTTC	CTCCCAGGGTAGAGGTGAAA
18.	*Bacteroidetes*	ACGCTAGCTACAGGCTTAACA	ACGCTACTTGGCTGGTTCA
19.	*Firmicutes*	GCGTGAGTGAAGAAGT	CTACGCTCCCTTTACAC
20.	*Lachnospiraceae*	CGGTACCTGACTAAGAAGC	AGTTTYATTCTTGCGAACG

## Supplementary Materials

The following supporting information can be downloaded at: https://www.mdpi.com/article/10.3390/ijms23179577/s1.
