# Supplementary material for: Intrarectal Capsazepine Administration Modulates Colonic Mucosal Health in Mice
Source: Int J Mol Sci. 2022 Aug 24;23(17):9577. doi: 10.3390/ijms23179577 (PMC9455796; doi:10.3390/ijms23179577)
Supplement: Supplementary file 1 [file ijms-23-09577-s001.zip › ijms-1812823-supplementary.pdf]

Supplementary Figures

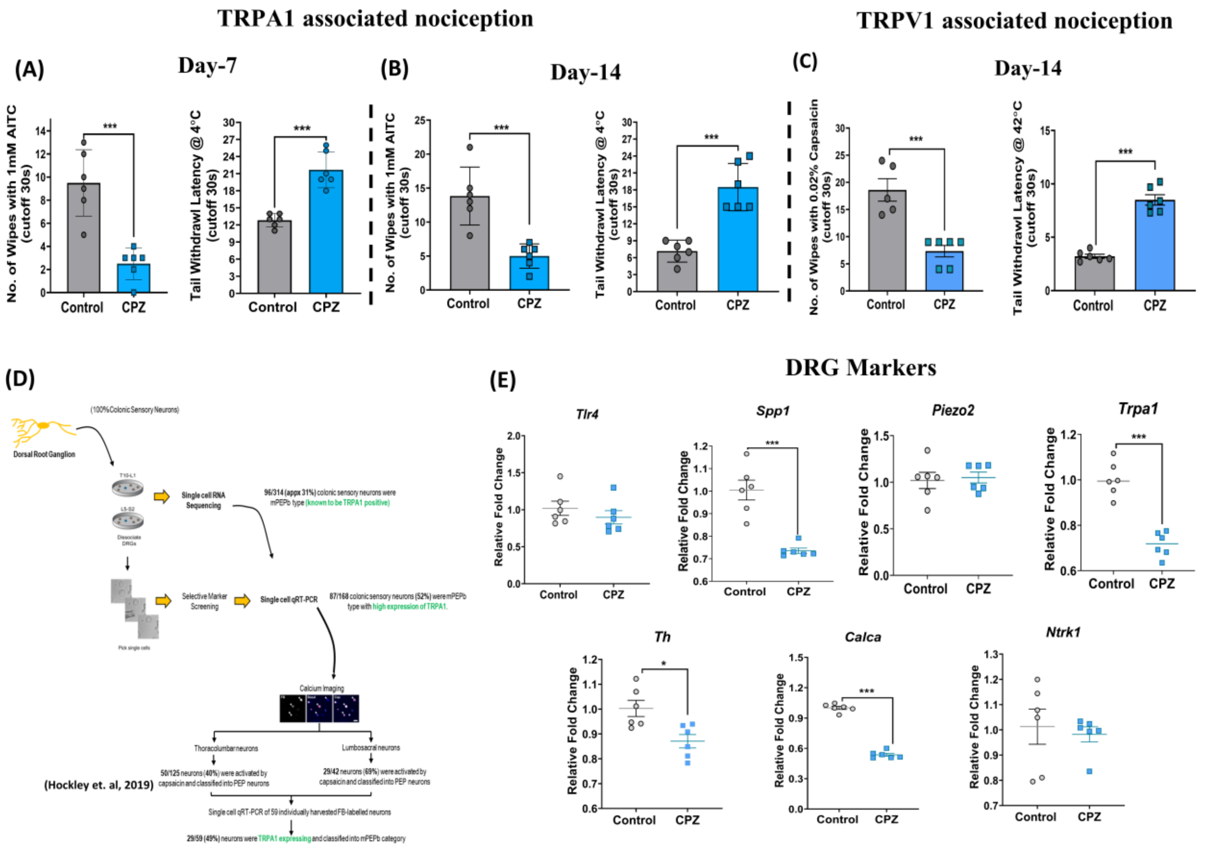

Supplementary Figure S1: Effect of CPZ rectal administration on TRPA1-induced nociceptive behavior and peptidergic sensory neuron markers on DRGs.

A) AITC-induced eye wipe test and test for tail withdrawal latency at 4ºC on day-7 (n=6). B) AITC induced eye wipe test and test for tail withdrawal latency at 4ºC on day-14 (n=6). C) Capsaicin-induced eye wipe test and tail withdrawal latency test at 42ºC on day-14 (n=6). D) Representative image for classification of TRPA1-expressing colonic sensory neurons. E) Gene expression for DRG-associated makers for peptidergic sensory neurons (n=3). Mice were divided into two groups – Control (administered vehicle rectally) and CPZ (administered 531µM CPZ rectally). Treatment was given for 2 weeks. Desensitization of TRPA1 nociceptive neurons was confirmed with AITC (1mM) - induced eye wipes and Tail withdrawal latency test at 4°C on day-7 and day-14 (n=6). Desensitization of TRPV1 sensory neurons was confirmed with Capsaicin (0.02% w/v) - induced eye wipes and Tail withdrawal

latency test at 42°C on day-14. After sacrifice, DRGs were harvested, RNA was isolated form DRGs and gene expression was performed for the markers of colonic peptidergic sensory neurons. All data is represented as mean ± SEM. Intergroup variations were assessed using Student unpaired t-test. *p<0.05, **p<0.01, *** p<0.001 versus Control.

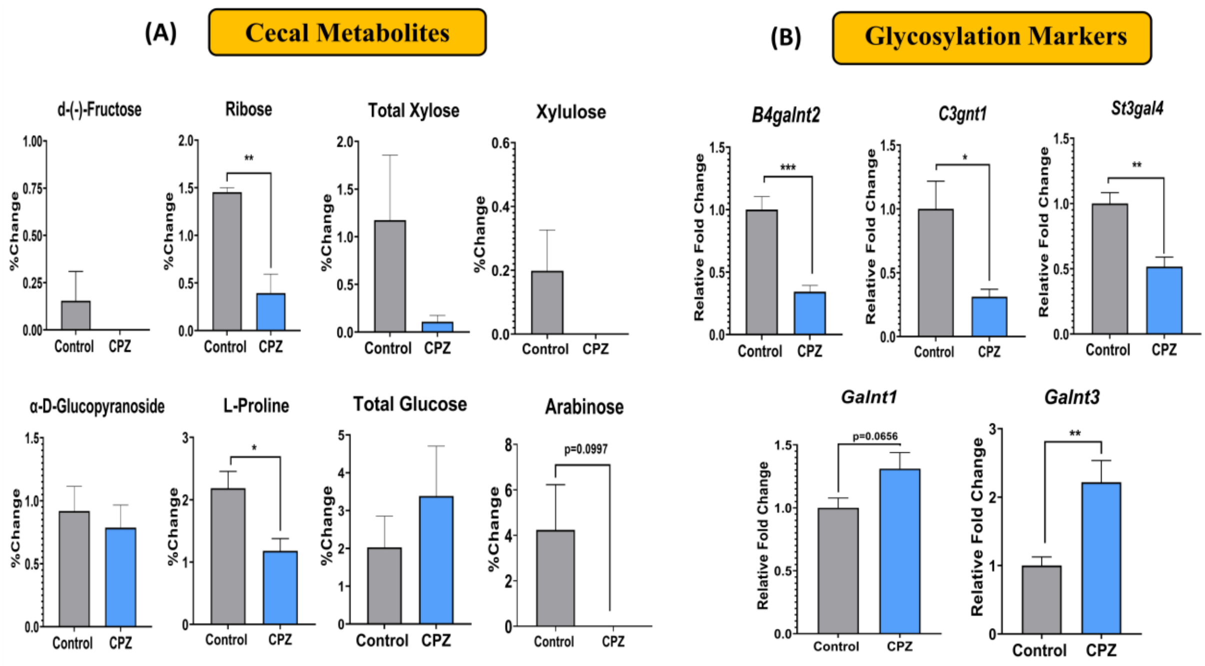

Supplementary Figure S2: Effect of CPZ rectal administration on mucin glycans and genes involved in mucus glycosylation.

A) Metabolite profiling of cecum content for mucin glycans using GC-MS (n=3). B) Expressional changes in genes involved in mucus glycosylation (n=6). Mice were divided into two groups – Control (administered vehicle rectally) and CPZ (administered 531µM CPZ rectally). Treatment was given for 2 weeks. Post-sacrifice, the cecum content was collected, weighed and treated overnight with methanol, chloroform and water in 2.5:1:1 ratio at 4ºC (n=3). Methanol and chloroform were evaporated and sample was lyophilized. The samples were lyophilized and derivatized using BSTFA+1%TMCS, pyridine (70°C, 4 h). Following, the sample was diluted with DCM and run in GCMS. For gene expression studies, RNA was extracted from colon tissues and gene expression was performed for mucus glycosylation genes using Nanostring nCounter multiplex gene expression assay (n=6). All data

is represented as mean ± SEM. Intergroup variations were assessed using Student unpaired t-test. *p<0.05, **p<0.01, *** p<0.001 versus Control.

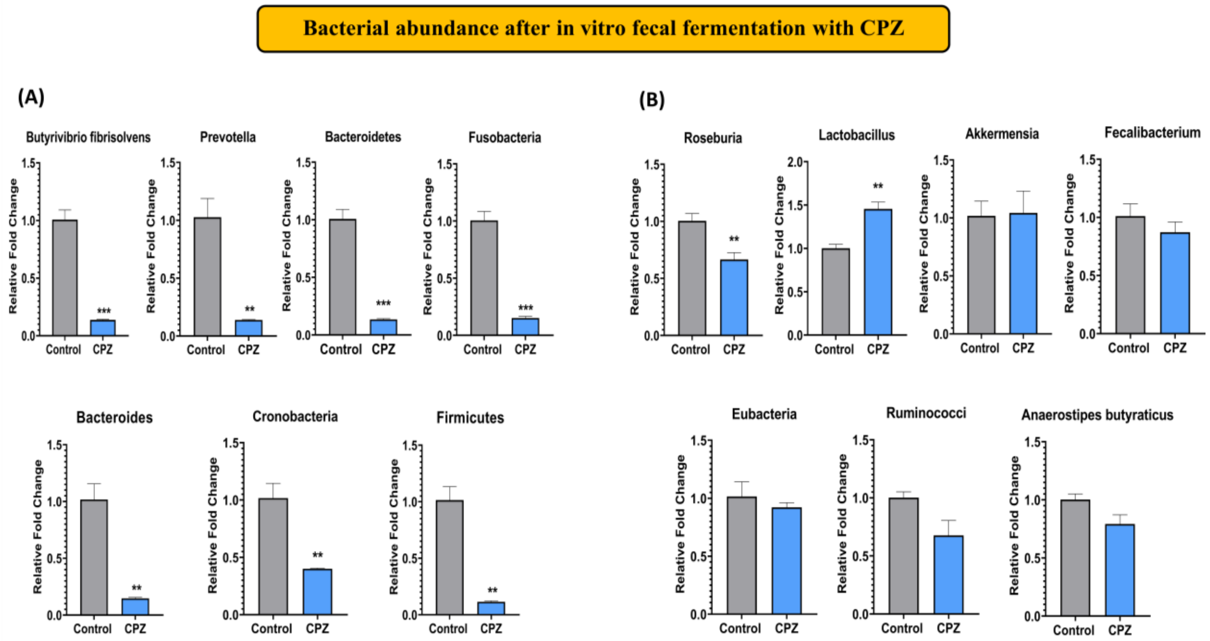

Supplementary Figure S3: Direct effect of CPZ on *in vitro* fermentation of gut microbiota.

A) Relative abundance of Gram -ve bacteria (after 48 h of fecal fermentation with or without 531µM CPZ treatment). B) Relative abundance of Gram +ve bacteria during *in vitro* fecal fermentation bacteria (after 48 h of fecal fermentation with without 531µM CPZ treatment). Briefly, fresh feces obtained from 12 normal C57BL/6J mice were pooled and a slurry of 0.1mg/ml was prepared in PBS. Cultures with 10X slurry dilution were prepared for: Control (treated with 1% w/v glucose) and treatment (treated with 531µM CPZ) groups (n=3). The cultures were incubated at 37°C under anaerobic conditions. After 48 h, the bacterial DNA was isolated from the culture using commercial kit. The DNA was subjected to qRT-PCR with primers of various bacterial genera. All data is represented as mean ± SEM. Intergroup variations were assessed using Student unpaired t-test. *p<0.05, **p<0.01, *** p<0.001 versus Control.

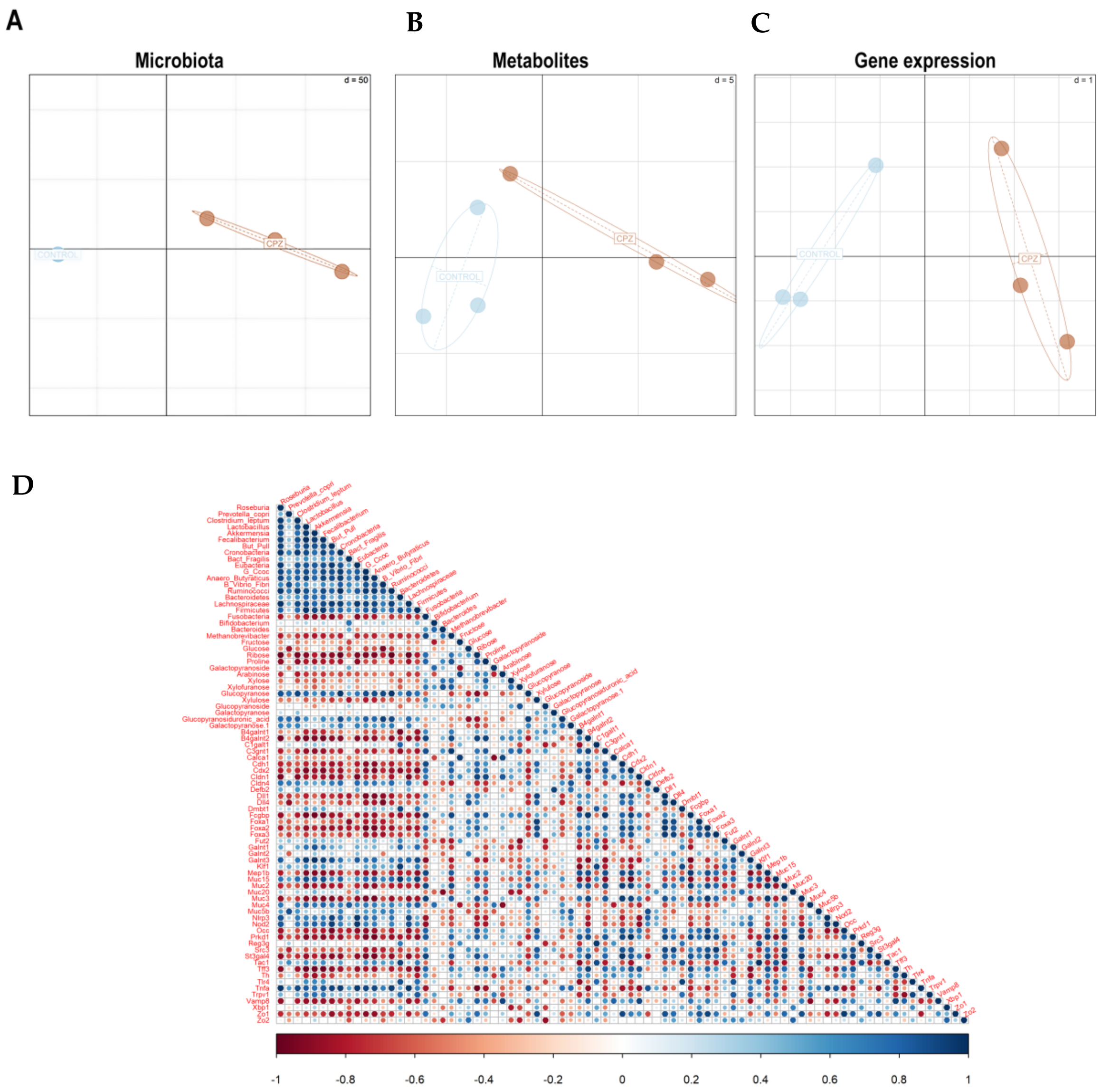

Supplementary Figure S4: Interaction between microbial population, metabolites and genes expression of Control and CPZ groups.

A) Principal component analysis (PCA) plot for gut microbiota interactions between Control and CPZ treated group. B) PCA plot for interaction between metabolites in Control and CPZ group. C) PCA plot for interaction between gene expression changes in Control and CPZ group. D) Correlation matrix between parameters assessed in the Control and CPZ group. Both PC and correlation analysis were carried out using the adegenet and corrplot packages in R.

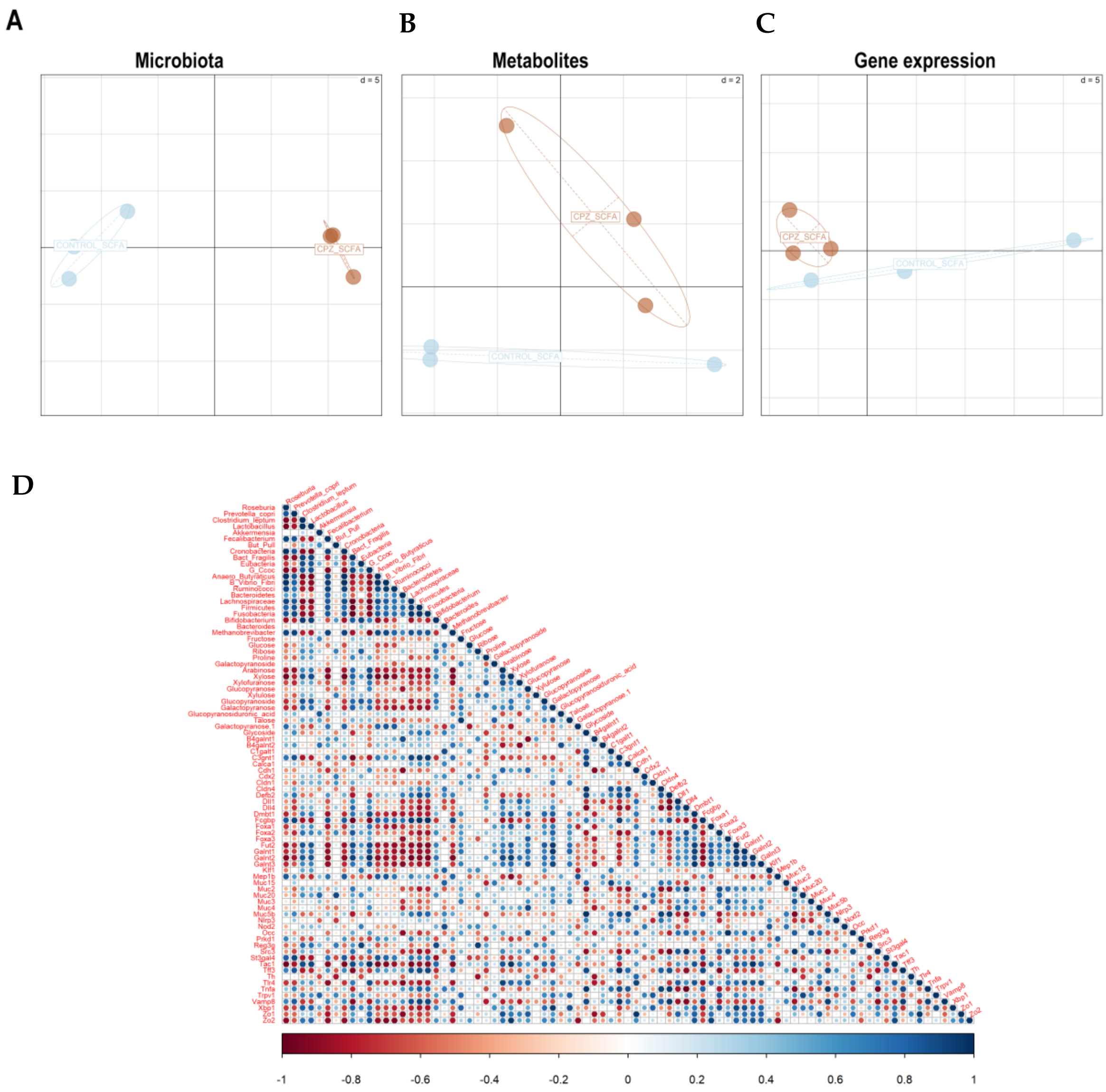

Supplementary Figure S5: Interaction between microbial population, metabolites and genes expression of SCFA and CPZ-SCFA groups.

A) Principal component analysis (PCA) plot for gut microbiota interactions between SCFA (Control-SCFA) and CPZ-SCFA treated group. B) PCA plot for interaction between metabolites in Control-SCFA and CPZ-SCFA group. C) PCA plot for interaction between gene expression changes in Control-SCFA and CPZ-SCFA group. D) Correlation matrix between parameters assessed in the Control-SCFA and CPZ-SCFA group. Both PC and correlation analysis were carried out using the adegenet and corrplot packages in R.
